# Eight-year follow-up of patient-reported outcomes in patients with breast cancer participating in exercise studies during chemotherapy

**DOI:** 10.1007/s11764-024-01640-0

**Published:** 2024-08-05

**Authors:** David Binyam, Willeke R. Naaktgeboren, Wim G. Groen, Neil K. Aaronson, Anouk E. Hiensch, Wim H. van Harten, Martijn M. Stuiver, Anne M. May

**Affiliations:** 1 Julius Center, University Medical Center Utrecht, Universiteitsweg 100, 3584CG Utrecht, The Netherlands; 2 Division of Psychosocial Research and Epidemiology, The Netherlands Cancer Institute, Amsterdam, The Netherlands; 3 Department of Medicine for Older People, Amsterdam UMC, Vrije Universiteit Amsterdam, Amsterdam, The Netherlands; 4 Aging & Later Life, Amsterdam Public Health Research Institute, Amsterdam, The Netherlands; 5 Amsterdam Movement Sciences, Ageing & Vitality, Rehabilitation & Development, Amsterdam, The Netherlands; 6 Department of Health Services and Technology Research, University of Twente, Enschede, The Netherlands; 7 Rijnstate Hospital, Arnhem, The Netherlands; 8 Faculty of Health, Amsterdam University of Applied Sciences, Amsterdam, The Netherlands

**Keywords:** Anxiety and depression, Breast cancer survivors, Fatigue, Physical activity, Long-term, Quality of life

## Abstract

**Purpose:**

Numerous randomized controlled trials (RCTs) have shown beneficial exercise effects on fatigue, anxiety and depression and health-related quality of life (HRQoL) in breast cancer (BC) patients during and shortly after treatment. Here, we investigated the long-term effects of exercise during chemotherapy for BC on these outcomes.

**Methods:**

We invited participants of two highly comparable RCTs that investigated the effects of exercise (EX) (versus usual care (UC)) during chemotherapy in patients with non-metastatic BC (*N* = 357) to participate in an 8-year follow-up. In both trials, fatigue, anxiety and depression and HRQoL were assessed using the same questionnaires, at multiple timepoints. Linear mixed-effect models were used to compare study arms over time.

**Results:**

In total, 156 participants (EX = 82; UC = 74) completed the follow-up questionnaires. EX reported comparable general (between-group difference 0.73, 95% confidence interval (− 0.35; 1.80), ES = 0.18) and physical fatigue (0.55 (− 0.55; 1.65), ES = 0.13), small but statistically significantly higher levels of anxiety (1.24 (0.47 to 2.00), ES = 0.39) and depression (1.10 (0.34; 1.85), ES = 0.38), significantly lower global HRQoL (− 5.99 (− 10.65; − 1.32), ES = 0.34) and comparable summary HRQoL (− 1.90 (− 4.70; 0.89), ES = 0.16) compared to UC.

**Conclusion:**

No long-term beneficial effects of exercise during chemotherapy on BC patients’ fatigue, anxiety, depression or HRQoL were observed. The less favourable outcomes for mood and HRQoL that were observed 8 years after participation in an exercise intervention may be explained by selective loss-to-follow-up.

**Implications for cancer survivors:**

The results highlight the need to incorporate strategies that promote physical activity maintenance after participation in an exercise programme to also counteract long-term detrimental side effects of cancer treatment.

**Supplementary Information:**

The online version contains supplementary material available at 10.1007/s11764-024-01640-0.

## Introduction

Exercise interventions for patients with breast cancer receiving chemotherapy have been studied extensively for their protective effects against treatment-related side effects [1, 2]. Beneficial effects have been observed for fatigue, but also for anxiety, depressive symptoms and health-related quality of life (HRQoL) [1–6]. However, there is only limited evidence regarding the long-term benefits of exercise, as the follow-up period of most studies does not surpass 2 years [2, 6, 7].

Exercise interventions during treatment can potentially have beneficial effects on long-term side effects through prevention of side effect development in the short term and through stimulation of long-term sustained increased physical activity levels. Indeed, of the few studies with a follow-up period surpassing 6 months, several have shown long-term effects of short exercise interventions during treatment on physical activity levels [7–9]. One study in early-stage breast cancer survivors reported beneficial effects of an exercise intervention (i.e. more leisure time physical activity and improved mood) at 18 and 60 months post-intervention [8]. In the 4-year follow-up study of the physical activity during cancer treatment (PACT) randomized controlled trial (RCT), significantly higher levels of total physical activity time and a trend towards less physical fatigue were reported by patients with breast cancer who were randomized to a supervised exercise programme during chemotherapy, as compared to the usual care control group [9]. Another RCT of a 12-month exercise programme after adjuvant treatment found that patients with breast cancer who had increased their physical activity level, irrespective of group allocation, had lower levels of fatigue and better HRQoL 5 years after inclusion [10]. However, in the same study, no association between group allocation and increased physical activity levels 5 years after inclusion was found [10]. These results underscore the possible beneficial effects of long-term increased physical activity levels which may be achieved via exercise programmes during chemotherapy treatment that are aimed at physical activity level maintenance. To our knowledge, there are no studies that have evaluated the extent to which the effects of exercise interventions during chemotherapy on cancer- and treatment-related fatigue, anxiety and depression and HRQoL are sustained beyond 5 years. Such information is important because the large majority of patients with non-metastatic breast cancer survive well past this milestone.

The PACT [11] and the Physical Exercise during Adjuvant Chemotherapy Effectiveness Study (PACES) [12] were both multi-centre RCTs, conducted in the Netherlands between 2010 and 2013. In these studies, patients with non-metastatic breast cancer were randomized to a supervised exercise programme during the period that they were undergoing treatment with chemotherapy. Both trials found beneficial effects of the exercise intervention on (among other outcomes) physical fitness and fatigue after chemotherapy completion. However, these effects diminished after 6 months, probably because the control group started exercising after the intervention period [9, 11, 12]. The combined follow-up study of these two trials (Pact-Paces-Heart) has as its primary objective the assessment of the potential long-term effects of exercise in protecting against cancer-related cardiotoxicity [13]. A secondary objective of that study, presented here, is the assessment of whether the supervised exercise interventions delivered, on average, 8 years earlier have lasting beneficial effects on fatigue, anxiety and depressive symptoms and HRQoL. Since the combined data from the PACT and PACES trials regarding the short-term effects on patient-reported outcomes (PROs) have not been published previously, this analysis has also been included in the current study.

## Methods

### PACT and PACES study designs

**Fig. 1:**
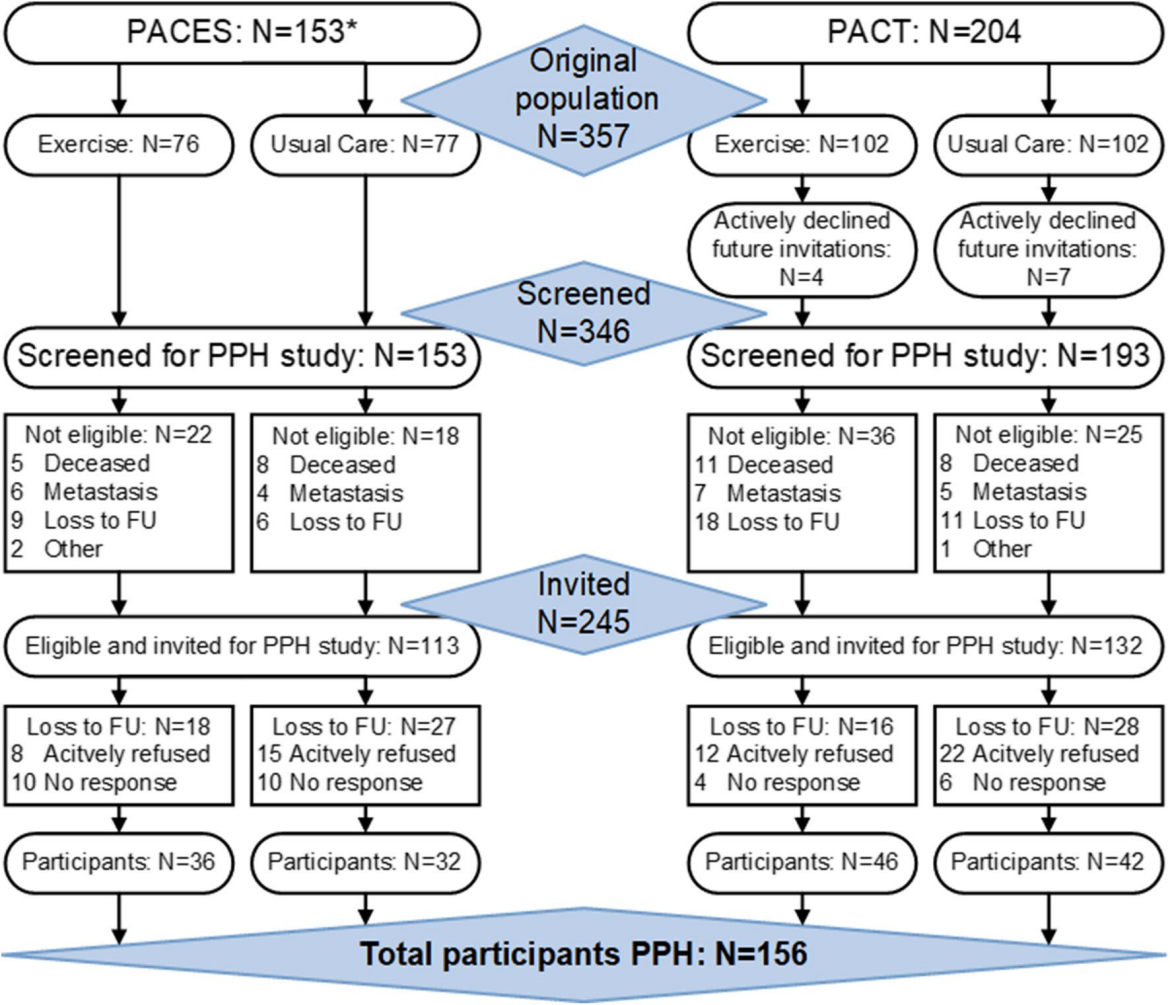
Flow chart of the patient flow of the Pact-Paces-Heart study. *=Participants who were randomized into the PACES Onco-Move intervention are not shown as they were excluded from the analyses in this paper

The design of both the PACT and PACES trials has been published previously [14–16]. Both trials were performed between 2009 and 2013 in the Netherlands and included patients with a histologically confirmed non-metastatic breast cancer diagnosis who were scheduled to receive chemotherapy. The most important exclusion criteria included any contra-indications for physical activity and not being able to read or understand Dutch [11, 12] (Online Resource 1 contains an overview of the complete in- and exclusion criteria). In the PACT trial (*N* = 204), recruited patients were randomized to (1) a moderate- to high-intensity, supervised exercise programme or (2) a usual care control group [11, 15]. In the PACES trial (*N* = 230), patients were randomized to (1) a moderate- to high-intensity, supervised exercise programme, comparable to the PACT study; (2) a home-based, low-intensity exercise programme (Onco-Move), which is excluded from the current analysis due to incomparability with the PACT study (*N* = 77); or (3) a usual care control group [11, 12]. The two trials combined thus included a total of 357 patients with breast cancer (PACT *N* = 204; PACES *N* = 153) (excluding PACES trial participants randomized in the Onco-Move group), of whom 178 were randomized to a moderate- to high-intensity, supervised exercise intervention (PACT *N* = 102; PACES *N* = 76) and 179 to usual care (PACT *N* = 102; PACES *N* = 77) (Fig. 1) [11, 12].

#### Exercise programme

Patients allocated to the supervised intervention arm in the PACT and PACES trials followed a moderate- to high-intensity, combined aerobic and resistance exercise programme, tailored to the participant’s fitness level and guided by a specialized physical therapist [11, 12, 14, 15]. In both trials, patients attended two supervised 1-h training sessions per week and were encouraged to be physically active for 5 days per week for at least 30 min (Online Resource 2). The intervention in the PACT trial incorporated principles of Bandura’s social cognitive theory (i.e. stimulating self-efficacy via mastery experience, observational experience and verbal persuasion) to encourage maintenance of physical activity after completion of the trial [15]. In the PACES trial, physical activity maintenance was stimulated through the use of the ‘active living’ method and the distribution of written information about physical activity tailored to the stage of change of participants [14]. Neither intervention included additional psychological support. Timing of the interventions differed slightly between the studies. In the PACT trial, participants exercised for 18 weeks, which overlapped, at least partly, with their chemotherapy. In the PACES trial, participants started the exercise programme at the first cycle of chemotherapy and continued until 3 weeks after the last cycle (on average 16 weeks). Patients in the control group in both studies received usual care as specified by hospital guidelines and were asked to maintain their regular physical activity level throughout the study period [9, 17–19]. Given that the PACT and PACES supervised exercise groups followed similar exercise programmes, their data were combined in the current analysis, as were the data from the two usual care control groups.

### Pact-Paces-Heart study

For the current follow-up study, all patients with breast cancer who participated in either the PACT or PACES trial and were still alive were eligible for recruitment. Participants were excluded if they were deemed ineligible by their treating physician (e.g. too mentally burdensome or severe neuropathy); had declined to be invited for future studies; were treated with chemo-, targeted, or thoracic radiotherapy for recurrent breast cancer; or were treated with systemic or thoracic radiotherapy for other malignancies than breast cancer (excluding non-melanoma skin cancer) after completion of the initial trial (Online Resource 1).

The participants in the 4-year follow-up PACT study [9] who had provided consent to be approached for future follow-up studies were, after screening for eligibility, directly approached by the research team for participation in the current study. The remaining original PACT participants, if they had not declined to be invited for future studies, and all PACES trial participants were approached by their treating physician. Eligible participants were invited for a study visit at the University Medical Centre Utrecht for informed consent procedures and outcome assessments including online questionnaires thereafter. The study was approved by the Medical Ethics Committee of the University Medical Centre Utrecht (METC 18/136).

#### Outcome measures

The primary endpoints of the Pact-Paces-Heart study were cardiovascular outcomes to assess the long-term cardiotoxicity of chemotherapy treatment and are reported in a separate paper [13]. The follow-up time of approximately 8 years was chosen to allow enough time for the development of possible cardiac damage. Here, we report on the simultaneously assessed PROs, including fatigue, anxiety and depression and HRQoL, together with the combined PRO results from earlier timepoints.

In the PACT trial, outcomes were assessed at baseline (T0), post-intervention (i.e. after 18 weeks, T1) and 36 weeks post-baseline (T2). Outcomes were assessed at similar timepoints in the PACES trial, namely at baseline (T0), after completing chemotherapy (T1) and 6 months after chemotherapy completion (T2). Additionally, in a 4-year post-baseline follow-up study, fatigue and HRQoL, but not anxiety and depression, were again assessed for 110 patients from the PACT trial (exercise *N* = 59; usual care *N* = 51) (T3) [9]. PROs were assessed using the same questionnaires as used in both the PACT and PACES trials (i.e. the Multidimensional Fatigue Inventory (MFI) for fatigue, the Hospital Anxiety and Depression Scale (HADS) for anxiety and depression and the European Organisation for Research and Treatment of Cancer (EORTC) Quality of Life Questionnaire (QLQ-C30) for HRQoL). For the analysis of fatigue, only the general and the physical fatigue subscales from the MFI were used, because of their reported reliability [20] and a hypothesized probable effect of exercise, respectively [11, 12].

In addition to measurements of PROs at the 8-year follow-up, physical activity levels were also assessed. The validated Short Questionnaire to Assess Health-enhancing physical activity (SQUASH) was used for this assessment [21].

#### Baseline characteristics

Sociodemographic data (i.e. sex, age and educational level), data regarding cancer (i.e. receptor status) and treatment characteristics (i.e. radiotherapy (yes/no)) were recorded at baseline in both the PACT and PACES trials via medical record examination. Physical activity levels at baseline were assessed via questionnaires, i.e. the SQUASH for PACT trial participants and the Physical Activity Scale for the Elderly in the PACES trial participants. Baseline characteristics for both the study sample that completed the 8-year follow-up for the Pact-Paces-Heart study and the sample that did not complete the follow-up will be presented, to allow for assessment for possible selective loss-to-follow-up.

### Statistical analyses

All statistical analyses were performed using R (version 4.2.2) and Rstudio (Version 2023.06.0; Rstudio Inc., Boston, MA). Baseline characteristics of the 8-year follow-up study sample and of those women from the original trials who did not participate in the follow-up study were summarized using descriptive statistics. For the HADS anxiety and depression scores, both mean values and categorical threshold values were calculated, with a score of 0–7 corresponding to ‘non-case’, 8–10 to ‘doubtful case’ and 11–21 to ‘probable case’ [22]. Frequencies of each category per treatment arm and timepoint were reported. To gain insight into fatigue and quality of life in the study sample, as compared to the general population, MFI and EORTC QLQ-C30 outcomes were compared to available reference values based on German and Dutch general population samples, respectively, stratified by age and sex [23, 24].

To analyse the potential short- and long-term effect of the exercise intervention on the PROs, we used intention-to-treat, linear mixed-effects models with a random intercept and unstructured covariance structure. Questionnaire scores from participants with data of at least two timepoints were entered in the model. We adjusted the models for education level (low, middle or high), age, initial study (PACT or PACES), tumour receptor status (triple negative; Her2Neu + and oestrogen receptor (ER) + or progesterone receptor (PR) + ; Her2Neu + ER/PR − ; Her2Neu − ER/PR +) and the respective baseline PRO scores. Time and group assignment were entered in the model both separately and as an interaction term. With the mixed models, marginal means for the questionnaire scores at all timepoints were estimated for the pooled exercise and usual care groups. Between-group differences at all post-baseline timepoints with corresponding 95% confidence intervals were calculated to assess any significant difference between the exercise and usual care groups. Standardized effect sizes (ESs) were calculated per timepoint by dividing the adjusted between-group differences by the pooled standard deviations at baseline. Using Cohen’s interpretation, ESs < 0.2 correspond to ‘no difference’, ESs between 0.2 and 0.5 to ‘small differences’, 0.5–0.8 to ‘moderate differences’ and ESs > 0.8 correspond to ‘considerable differences’ [25].

To assess selective loss-to-follow-up in the combined study sample, mean PRO scores at baseline, T1 and T2 were compared between the groups that were successfully recruited into the Pact-Paces-Heart study and the groups that were not. The statistical significance of the difference between the means was assessed with unpaired *t*-tests. For all analyses, *p*-values smaller than 0.05 were considered statistically significant.

## Results

### Participants

In total, 346 of the 357 participants of the PACT (*N* = 193) and the PACES trial (*N* = 153) randomized to either the supervised exercise intervention or the control group were successfully screened for eligibility between September 2018 and November 2021. Overall, 245 participants (70.8% of screened participants) were invited to participate in the follow-up study (Fig. 1). Ineligibility was primarily due to death (9.2%), having metastases (6.4%) or having been lost to follow-up (12.7%). Of the 245 invited participants, 57 (23.3%) actively declined to participate and 32 (13.1%) did not respond, resulting in a total sample of 156 (63.7%). Of these participants, 82 had been assigned to the supervised exercise intervention and 74 had received usual care during their chemotherapy treatment (Fig. 1).

Baseline characteristics of the exercise and control group included in the follow-up study were largely comparable (Table 1). All included participants were female with a mean age of 50.3 (SD = 7.2) at baseline. Regarding tumour receptor status, the control group included more HER − ER/PR + (68.9% vs 53.7%) and fewer triple negative (12.2% vs 19.5%) tumours compared to the exercise group. The mean follow-up time was 8.5 years (SD = 1.1) (exercise 8.4 (1.2); usual care 8.6 (1.1)). When comparing the follow-up study sample with the PACT and PACES trial sample lost to follow-up, the follow-up sample had a somewhat higher educational level at baseline for both the exercise and control groups. The participants in the follow-up study drawn from the PACT trial had higher levels of baseline physical activity compared to the PACT participants not recruited into the follow-up study (Table 1).

**Table 1:** Baseline characteristics of the PACT and PACES trial participants that were included in the PACT-PACES-HEART study and of those who were lost to follow-up

	Included in Pact-Paces-Heart			Lost to follow-up		
	All participants (*N* = 156)	Control (*N* = 74)	Intervention (*N* = 82)	All participants (*N* = 201)	Control (*N* = 105)	Intervention (*N* = 96)
Sex						
Male	0 (0%)	0 (0%)	0 (0%)	2 (1.0%)	0 (0%)	2 (2.1%)
Female	156 (100%)	74 (100%)	82 (100%)	199 (99.0%)	105 (100%)	94 (97.9%)
Age						
Mean (SD)	50.3 (7.22)	49.9 (7.23)	50.7 (7.23)	49.9 (9.06)	50.7 (9.04)	49.0 (9.04)
Educational level						
Low	8 (5.1%)	6 (8.1%)	2 (2.4%)	26 (12.9%)	19 (18.1%)	7 (7.3%)
Medium	59 (37.8%)	28 (37.8%)	31 (37.8%)	88 (43.8%)	47 (44.8%)	41 (42.7%)
High	88 (56.4%)	39 (52.7%)	49 (59.8%)	79 (39.3%)	34 (32.4%)	45 (46.9%)
Missing	1 (0.6%)	1 (1.4%)	0 (0%)	8 (4.0%)	5 (4.8%)	3 (3.1%)
Initial trial						
PACT	88 (56.4%)	42 (56.8%)	46 (56.1%)	116 (57.7%)	60 (57.1%)	56 (58.3%)
PACES	68 (43.6%)	32 (43.2%)	36 (43.9%)	85 (42.3%)	45 (42.9%)	40 (41.7%)
Radiotherapy						
No	39 (25.0%)	20 (27.0%)	19 (23.2%)	55 (27.4%)	29 (27.6%)	26 (27.1%)
Yes	117 (75.0%)	54 (73.0%)	63 (76.8%)	146 (72.6%)	76 (72.4%)	70 (72.9%)
Tumour receptor status						
Triple negative	25 (16.0%)	9 (12.2%)	16 (19.5%)	41 (20.4%)	20 (19.0%)	21 (21.9%)
HER + ER/PR +	25 (16.0%)	11 (14.9%)	14 (17.1%)	34 (16.9%)	22 (21.0%)	12 (12.5%)
HER + ER/PR −	11 (7.1%)	3 (4.1%)	8 (9.8%)	6 (3.0%)	2 (1.9%)	4 (4.2%)
HER − ER/PR +	95 (60.9%)	51 (68.9%)	44 (53.7%)	120 (59.7%)	61 (58.1%)	59 (61.5%)
Moderate/vigorous total PA at baseline (min/week)^a^						
Median (IQR)	600 (337.5, 1172.8)	735 (370, 1305)	522.5 (300, 1140)	510 (215, 1225)	595 (277.5, 1447.5)	420 (210, 892.5)
PASE score at baseline^b^						
Median (IQR)	75.3 (47.9, 113.6)	64.4 (40.6, 106.9)	79.5 (49.4, 119.4)	80 (44.5, 148.9)	84.3 (44.7, 183.7)	74.3 (41.7, 120.8)

^a^Only measured for participants originating from the PACT trial, measured using the Short Questionnaire to Assess Health-enhancing physical activity

^b^Only measured for the participants originating from the PACES trial

Abbreviations: *ER* oestrogen receptor, *HER* human epidermal growth factor receptor 2, *PA* physical activity, *PASE* Physical Activity Scale for the Elderly, *PR* progesteron receptor, *SD* standard deviation

Physical activity levels at the final follow-up were comparable between the exercise and control group, with a median minutes/week of moderate- to high-intensity leisure and sport physical activity of 150 (IQR (60–368)) and 155 (IQR (60–360)), respectively.

### Main outcomes

#### Fatigue

**Fig. 2:**
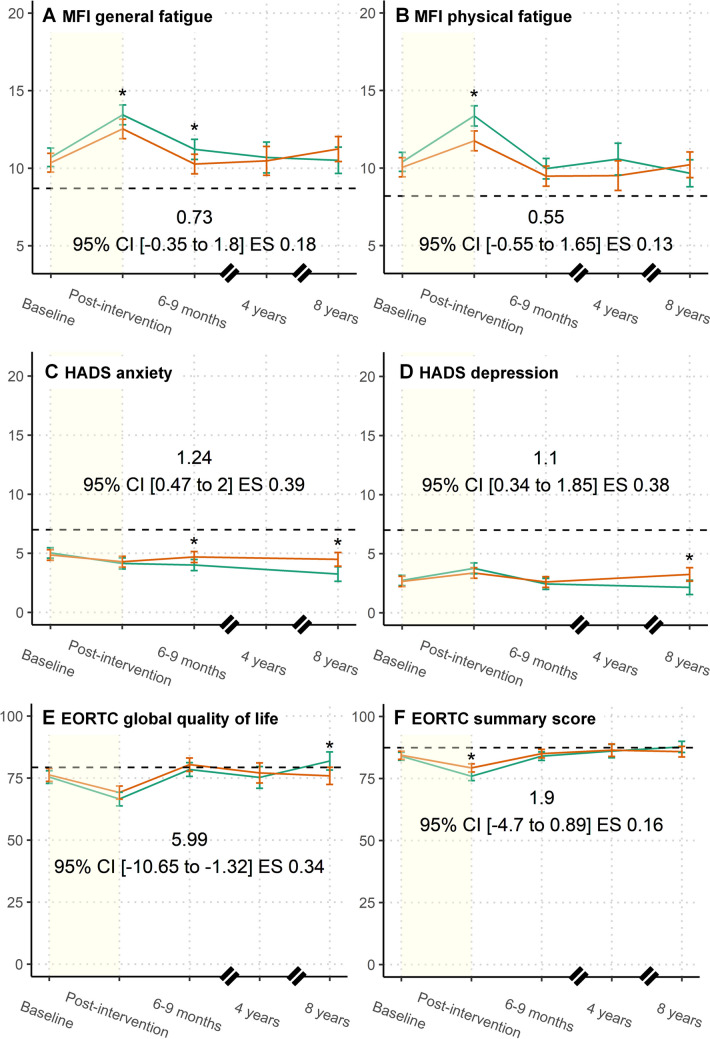
Effects of performing an exercise intervention during chemotherapy on patient-reported fatigue, anxiety, depression and quality of life. Intention-to-treat mixed-effect models were performed with all available information for the pooled groups that performed a supervised exercise intervention (orange) and the pooled usual care groups (blue) from the PACT and PACES trial. Models were used to calculate marginal means for **A** general fatigue and **B** physical fatigue assessed via the MFI, **C** anxiety and **D** depression as assessed via the HADS and the **E** global quality of life and **F** summary score from the EORTC QLQ-C30. Models were adjusted for education level, age, initial study, tumour receptor status and respective baseline outcome scores. Between-group differences were calculated, and the differences after 8-year follow-up are depicted on the figures with a 95% confidence interval and effect size. Where available, the dotted black lines represent either the reference value for age and sex-matched general population (MFI general fatigue = 8.7; physical fatigue = 8.2 and EORTC global HRQoL = 79.3; summary score = 87.4) or the threshold values (HADS, threshold for borderline case = 7) [22–24]. The yellow filled area represents the intervention period. *=Significant between-group difference (i.e. *p* < 0.05). Abbreviations: EORTC, European Organisation for Research and Treatment of Cancer; ES, standardized effect size; GP, general population; HADS, Hospital Anxiety and Depression Scale; MFI, Multidimensional Fatigue Inventory

For the general and physical fatigue subscales of the MFI, both the exercise and control groups had, on average, worse scores directly post-intervention compared to baseline (Fig. 2A, B; Table 2). Over the longer post-intervention period, the mean fatigue scores tended to return to around baseline levels. Compared to reference values for women between 40 and 59 years of age in the general population [23], participants scored higher for both general and physical fatigue at baseline and during the whole study period (Fig. 2A, B).

**Table 2:** Estimated marginal means of patient-reported outcomes at baseline, post-intervention, after 6–9 months, after ~ 4 years and after ~ 8 years, with between-group differences for the last timepoint

Measure	Baseline EMM (SE)	Post-intervention EMM (SE)	6–9 months EMM (SE)	4 years EMM (SE)	8 years EMM (SE)	Between-group difference T4	
						AMD (95% CI)	ES
General fatigue							
Control	10.7 (± 0.31)	13.4 (± 0.32)	11.2 (± 0.33)	10.7 (± 0.51)	10.5 (± 0.43)	0.73 (− 0.35; 1.80)	0.18
Exercise	10.4 (± 0.31	12.5 (± 0.32)	10.3 (± 0.32)	10.5 (± 0.48)	11.2 (± 0.41)		
Physical fatigue							
Control	10.4 (± 0.31)	13.4 (± 0.33)	10.0 (± 0.34)	10.6 (± 0.52)	9.7 (± 0.44)	0.55 (− 0.55; 1.65)	0.13
Exercise	10.1 (± 0.32)	11.7 (± 0.33)	9.5 (± 0.33)	9.5 (± 0.49)	10.2 (± 0.42)		
Anxiety							
Control	5.0 (± 0.22)	4.1 (± 0.24)	4.0 (± 0.24)	-	3.3 (± 0.31)	**1.24 (0.47; 2.00)**	**0.39**
Exercise	4.9 (± 0.23)	4.3 (0.23)	4.7 (± 0.24)	-	4.5 (± 0.30)		
Depression							
Control	2.7 (± 0.22)	3.8 (± 0.23)	2.4 (± 0.24)	-	2.2 (± 0.31)	**1.10 (0.34; 1.85)**	**0.38**
Exercise	2.7 (± 0.22)	3.4 (± 0.23)	2.6 (± 0.23)	-	3.2 (± 0.29)		
Global quality of life							
Control	75.4 (± 1.30)	66.6 (± 1.40)	78.5 (± 1.41)	75.3 (± 2.22)	81.9 (± 1.87)	− **5.99 (**− **10.65;** − **1.32)**	**0.34**
Exercise	76.3 (± 1.32)	69.2 (± 1.35)	80.4 (± 1.37)	77.1 (± 2.07)	75.9 (± 1.77)		
Summary quality of life							
Control	84.0 (± 0.82)	75.9 (± 0.88)	84.0 (± 0.88)	86.0 (± 1.37)	87.7 (± 1.13)	− 1.90 (− 4.70; 0.89)	0.16
Exercise	84.3 (± 0.83)	79.3 (± 0.85)	85.0 (± 0.86)	86.4 (± 1.27)	85.8 (± 1.09)		

Between-group differences with bold font were significant (i.e. *p* < 0.05). Anxiety and depression were not measured at 4-year follow-up

Abbreviations: *AMD* adjusted mean difference, *CI* confidence interval, *EMM* estimated marginal means, *ES* effect size, *SE* standard error

Post-intervention, the general fatigue scores were significantly lower for the exercise group compared to the control group, with a between-group difference of − 0.91 (95% CI (− 1.68; − 0.14), ES = 0.22). Similarly, the exercise group scored significantly lower than the control group for physical fatigue, post-intervention (− 1.61, 95% CI (− 2.40; − 0.82), ES = 0.37). For general fatigue, this significant between-group difference remained 6 to 9 months post-baseline (− 0.95, 95% CI (− 1.74; − 0.16), ES = 0.23), whereas for physical fatigue, it was no longer statistically significant at this timepoint (− 0.48, 95% CI (− 1.29; 0.33), ES = 0.11). Approximately 8 years post-baseline, no statistically significant differences were found between the exercise and control groups for either general fatigue (0.73, 95% CI (− 0.35; 1.80), ES = 0.18) or physical fatigue (0.55, 95% CI (− 0.55; 1.65), ES = 0.13).

#### Anxiety and depression

Immediately post-intervention, both the exercise and control groups reported somewhat lower anxiety and higher depression scores compared to baseline (Fig. 2C, D; Table 2). The study sample, on average, scored below the threshold for ‘doubtful cases’ and within the ‘non-case’ ranges during the whole study period (Fig. 2C, D) [22]. No significant differences between the exercise and control groups were found for the anxiety scores directly post-intervention (0.15, 95% CI (− 0.41; 0.78), ES = 0.05) and for the depression scores directly post-intervention (− 0.40, 95% CI (− 0.94; 0.15), ES =  − 0.14) and 6 to 9 months post-baseline (0.16, 95% CI (− 0.40; 0.71), ES = 0.06) (Fig. 2C, D). For anxiety, the intervention group had significantly elevated scores compared to the control group 6 to 9 months post-baseline (0.68, 95% CI (0.11; 1.24), ES = 0.21) (Fig. 2C). At 8-year follow-up, for both anxiety and depression scales, the intervention group scored significantly higher than the control group (1.24, 95% CI (0.47; 2.0), ES = 0.39 and 1.10, 95% CI (0.34; 1.85), ES = 0.38, respectively) (Fig. 2C, D; Table 2). However, the percentage of participants who were categorized as (borderline) anxiety/depression cases in the exercise group (15.9%/9.7%) was comparable to the control groups (10.9%/8.1%) after 8-year follow-up and over time (Online Resource 3 & 4).

#### Quality of life

For both the exercise and control groups, global QoL and the QLQ-C30 summary score declined directly post-intervention compared to baseline and dropped further below reference values for Dutch women of 50 to 59 years of age [24]. At subsequent timepoints, the scores recovered and exceeded baseline levels, returning to a level comparable to the reference values (Fig. 2E, F; Table 2). For global QoL, exercise and control groups showed no significant differences immediately post-intervention (2.61, 95% CI (− 0.72; 5.94), ES = 0.15).

The QLQ-C30 summary score was significantly higher in the exercise group directly post-intervention compared to the control group (3.38, 95% CI (1.33; 5.43), ES = 0.28). After longer follow-up, the exercise and control groups initially showed no significant difference in global QoL (1.93, 95% CI (− 1.48; 5.33), ES = 0.11, after 6 to 9 months); however, after 8-year follow-up, global QoL was significantly lower in the exercise groups compared to the control groups (− 5.99, 95% CI (− 10.65; − 1.32), ES = 0.34). The initial difference in QLQ-C30 summary score immediately post-baseline declined after longer follow-up (1.03, 95% CI (− 1.05; 3.11), ES = 0.09, after 6 to 9 months), and at 8-year follow-up, there was no longer a significant between-group difference (− 1.90, 95% CI (− 4.70; 0.89), ES = 0.16).

#### Selective loss-to-follow-up

Comparison of baseline, post-intervention and 6- to 9-month follow-up PRO scores of the participants in the original trials who were lost to follow-up versus those successfully recruited into the current 8-year follow-up study revealed a pattern for the fatigue subscales only. Those in the control group who did not participate in the 8-year follow-up study had higher scores for general and physical fatigue (Online Resource 5), with the difference reaching statistical significance for general fatigue at the immediate post-intervention assessment (− 1.34, 95% CI (0.02; 2.7), *p* = 0.047).

## Discussion

Exercise has been established as a promising strategy for reducing treatment-related side effects of adjuvant chemotherapy for breast cancer in the short term. The present Pact-Paces-Heart follow-up study assessed long-term effects (i.e. on average, 8 years after chemotherapy completion) of a supervised exercise programme during chemotherapy on fatigue, anxiety and depression and HRQoL in patients with breast cancer. Although beneficial effects of exercise were found on fatigue and HRQoL in the short term (i.e. immediately post-intervention), these effects were no longer observed approximately 8 years after chemotherapy completion. In fact, at that timepoint, the exercise group reported statistically significantly higher levels of anxiety, depression and a lower HRQoL than the control group, although standardized effect sizes were small (ranging from 0.34 for global HRQoL to 0.38 and 0.39 for depression and anxiety, respectively). 

When comparing the outcomes to reference and threshold values, the current study sample reported slightly higher fatigue scores than the general population. In contrast, participants scored, on average, well below the threshold values for ‘borderline’ anxiety and depression during the whole study period, and reported HRQoL comparable to the general population for a large part of the follow-up period [24]. The generally good scores on the questionnaires are in line with the higher educational level of the current sample, which is associated with higher self-reported health [26]. Furthermore, a relatively healthy and more highly educated study population is often seen in exercise trials, as these patients are more often willing to start or continue exercising [27]. Still, the relatively high scores for fatigue compared to a sample of the German general population can be expected for cancer patients. It must be noted, however, that due to the lack of availability of Dutch reference values, a truly correct comparison for the fatigue scores is hampered [23].

In line with our results, a recently published follow-up study of two RCTs reported no significant difference in self-reported fatigue and HRQoL in patients with breast cancer who had participated in either a supervised aquatic or unsupervised online exercise programme approximately 5 years earlier compared to patients who had received usual care [28]. Additionally, poor maintenance of physical activity levels was observed, as two-thirds of the women who had participated in the exercise programme were inactive (i.e. < 7.5 metabolic equivalent of task-hour/week) 5 years later [28]. The problem of poor maintenance of increased physical activity levels was also reported in a recent systematic review and meta-analysis [7]. Physical activity levels were moderately increased up to the first 6 months after completion of the programmes but decreased during the extended follow-up period (up to a maximum of 60 months) [7].

Assuming a beneficial effect of initially increased physical activity levels on fatigue, anxiety and depression and HRQoL, this trend over time would also be in line with the results of the current study, showing an initial benefit of the intervention, but no such benefit in the long term. This could be explained, at least in part, by a change in contrast in physical activity levels between the exercise and control groups at follow-up in the current study, with comparable physical activity levels in both treatment arms after 8-year follow-up. Several factors, augmented by the relatively long follow-up period compared to the duration of the exercise programmes, may underlie this change. First, it may reflect the non-maintenance of increased physical activity levels for some of the participants in the intervention group. Second, some control group patients may have started exercising during the period of active treatment, as was documented in the PACT trial [11], or after completing their chemotherapy. Unsolicited increases in physical activity levels in control group members, especially in a motivated population such as ours, is a common issue in lifestyle intervention trials [29]. Importantly, the follow-up period of 8 years in this study was well suited for the assessment of the primary cardiac outcomes but may have led to dilution of contrast between the study groups, for reasons unrelated to the intervention under study. Future studies are needed to investigate the effectiveness of exercise programmes that extend over a longer period and that include more elaborate cognitive behavioural intervention components that provide participants with the necessary tools to maintain their increased level of physical exercise after trial completion [30].

Our finding that the exercise group reported more symptoms of anxiety and depression and lower HRQoL than the control group at 8-year follow-up was unexpected. At the immediate post-intervention assessment, we found positive exercise effects on fatigue and HRQoL and no effect on anxiety or depression. In the pooled analyses, anxiety was already slightly, but statistically significant, higher in the exercise group 6 to 9 months post-baseline. This trend over time has not been reported previously in the literature. The higher anxiety scores observed in the exercise group in the period shortly after completing the exercise programme might reflect the immediate effects of the sudden loss of the support that the supervised exercise programme had provided. This would suggest the need for additional educational components that would help participants to transition to a maintenance exercise regimen in the long term. The higher level of anxiety and depression and lower HRQoL observed in the intervention group after the 8-year follow-up may also reflect decreased physical activity levels of the intervention group during the follow-up period. Due to the absence of information on physical activity levels during follow-up, however, we could not investigate this. Finally, the poorer outcomes for the intervention group at the final timepoint could also reflect some form of selective loss-to-follow-up in which the control group participants with better (and/or the intervention group participants with poorer) self-reported outcomes were more inclined to participate in the long-term follow-up assessment. Although no evidence of selective loss-to-follow-up was found for these PROs when comparing scores at baseline, post-intervention and at 6 to 9 months post-baseline between participants lost to follow-up and those who completed the follow-up, selection cannot be ruled out without data on PROs 8 years post-baseline in the group lost to follow-up. Importantly, at long-term follow-up, there were no significant differences observed between the intervention and control group in the percentage scoring above the threshold for borderline or probable cases for anxiety or depression, and both groups had comparable scores for HRQoL compared to normative data for the Dutch female general population.

Limitations of this study include the lack of detailed and reliable information on exercise behaviour during the full 8 years of follow-up and our inability to compare the responders and non-responders to the exercise intervention in this regard. Further limits of the study are the incomplete participation in the 8-year follow-up as well as the lack of detailed current information on the PACT and PACES participants who did not complete the follow-up. This may have resulted in bias due to possible selective loss-to-follow-up, although comparisons at earlier timepoints between the follow-up and loss-to-follow-up group show no clear signs of such bias. A strength of this follow-up study is the increased sample size obtained by combining the data of two previous trials, which was possible thanks to the strong similarity between the protocols of the original trials. Indeed, analyses stratified for the original trial showed no important differences, justifying the pooling of the two trials (data not shown).

In conclusion, in this 8-year follow-up study, we found short-term but no long-term, positive effects of two comparable exercise programmes implemented during adjuvant chemotherapy treatment for breast cancer, compared to the control arms. Surprisingly, we found elevated scores for anxiety and depression and lower HRQoL in the intervention group compared to the control group after an 8-year follow-up. We cannot entirely rule out the possibility that the null and negative results may reflect some mechanism of selective loss-to-follow-up in the study sample. Future research is needed to determine if an exercise programme that extends well into the survivorship period and that has a strong behaviour maintenance component can yield more lasting effects on targeted patient-reported outcomes.

## Supplementary Information

Below is the link to the electronic supplementary material.

Supplementary file1 (PDF 176 kb)

Supplementary file2 (PDF 123 kb)

Supplementary file3 (PDF 139 kb)

Supplementary file4 (PDF 264 kb)

Supplementary file5 (PDF 322 kb)

## Data Availability

The datasets generated during and/or analysed during the current study are not publicly available due to privacy reasons but are available from the corresponding author after contact via e-mail.
