# Supplementary material for: Eight-year follow-up of patient-reported outcomes in patients with breast cancer participating in exercise studies during chemotherapy
Source: J Cancer Surviv. 2024 Aug 5;20(1):123–33. doi: 10.1007/s11764-024-01640-0 (PMC12906584; doi:10.1007/s11764-024-01640-0)
Supplement: Supplementary file 1 — Supplementary file1 (PDF 176 kb) [file 11764_2024_1640_MOESM1_ESM.pdf]

Online Resource 1

Article name

8-year follow-up of patient-reported outcomes in patients with breast cancer participating in exercise studies during chemotherapy

Journal

Journal of Cancer Survivorship

Authors & affiliations

David Binyam[1]/Willeke R. Naaktgeboren[1,2] (shared first), Wim G. Groen[3,4,5], Neil K. Aaronson[2], Anouk E. Hiensch[1], Wim H. van Harten[2,6,7], Martijn M. Stuiver[2,8]/Anne M. May[1] (shared last)

1. University Medical Center Utrecht, The Netherlands; 2. Division Of Psychosocial Research and Epidemiology, The Netherlands Cancer Institute, Amsterdam, The Netherlands; 3. Department of Medicine for Older People, Amsterdam UMC, Vrije Universiteit Amsterdam, Amsterdam, The Netherlands; 4. Aging & Later Life, Amsterdam Public Health Research Institute, Amsterdam, The Netherlands; 5. Amsterdam Movement Sciences, Ageing & Vitality, Rehabilitation & Development, Amsterdam, The Netherlands. 6. Department of Health Services and Technology Research, University of Twente, Enschede, The Netherlands; 7. Rijnstate Hospital, Arnhem, The Netherlands; 8. Faculty of Health, Amsterdam University of Applied Sciences, Amsterdam, The Netherlands.

Corresponding author

Anne M. May, Universiteitsweg 100, 3584CG, Utrecht, The Netherlands;

E-mail: a.m.may@umcutrecht.nl

Phone number: +31887551132

Online Resource 1. In- and exclusion criteria of the PACT, PACES and Pact-Paces-Heart study

	PACT	PACES	Pact-Paces-Heart
**Inclusion criteria**	- Histologically confirmed non-metastatic breast cancer diagnosis less than six weeks ago<br>- Scheduled to receive adjuvant chemotherapy<br>- Patients with immediate us of tissue expander after surgery were included until ten weeks after histological diagnosis and after replacement of the tissue expander with a breast prothesis<br>- Age 25-75 years	- Histologically confirmed non-metastatic breast cancer diagnosis<br>- Scheduled to receive adjuvant chemotherapy	- Patients with breast cancer who had participated in either the PACT or the PACES trial
**Exclusion criteria**	- Having received treatment for (non-basal skin) cancer in the five years preceding recruitment<br>- Unable to read and understand Dutch<br>- Karnovsky Performance status of ≤ 60<br>- Unable to walk ≥ 100 meter<br>- Any contra-indication for physical activity as assessed through the Revised Physical Activity Readiness Questionnaire.	- The presence of any comorbidities that are a contraindication for participation in an exercise program, including:<br>o Serious orthopaedic conditions hampering functional recovery<br>o Serious cardiovascular or cardiopulmonary conditions (or risks) that do not allow for training at the required intensity level<br>o Serious psychiatric or cognitive problems<br>- Patients with a BMI < 18kg/m^2, unintended weight loss of more than 5% per month or more than 10% weight loss during the previous six months<br>- Unable to read and understand Dutch<br>- Participation in concurrent studies or rehabilitation programs containing exercise elements	- Death during follow-up<br>- Deemed ineligible by their treating physician (e.g., too mentally burdensome or severe neuropathy)<br>- Treated with chemo-, targeted, or thoracic radiotherapy for recurrent breast cancer during follow-up<br>- Treated with systemic or thoracic radiotherapy for other malignancies than breast cancer (excluding non-melanoma skin cancer) during follow-up<br>- Declined to be invited for future studies
