# Supplementary material for: Eight-year follow-up of patient-reported outcomes in patients with breast cancer participating in exercise studies during chemotherapy
Source: J Cancer Surviv. 2024 Aug 5;20(1):123–33. doi: 10.1007/s11764-024-01640-0 (PMC12906584; doi:10.1007/s11764-024-01640-0)
Supplement: Supplementary file 2 — Supplementary file2 (PDF 123 kb) [file 11764_2024_1640_MOESM2_ESM.pdf]

Online Resource 2

Article name

8-year follow-up of patient-reported outcomes in patients with breast cancer participating in exercise studies during chemotherapy

Journal

Journal of Cancer Survivorship

Authors & affiliations

David Binyam[1]/Willeke R. Naaktgeboren[1,2] (shared first), Wim G. Groen[3,4,5], Neil K. Aaronson[2], Anouk E. Hiensch[1], Wim H. van Harten[2,6,7], Martijn M. Stuiver[2,8]/Anne M. May[1] (shared last)

1. University Medical Center Utrecht, The Netherlands; 2. Division Of Psychosocial Research and Epidemiology, The Netherlands Cancer Institute, Amsterdam, The Netherlands; 3. Department of Medicine for Older People, Amsterdam UMC, Vrije Universiteit Amsterdam, Amsterdam, The Netherlands; 4. Aging & Later Life, Amsterdam Public Health Research Institute, Amsterdam, The Netherlands; 5. Amsterdam Movement Sciences, Ageing & Vitality, Rehabilitation & Development, Amsterdam, The Netherlands. 6. Department of Health Services and Technology Research, University of Twente, Enschede, The Netherlands; 7. Rijnstate Hospital, Arnhem, The Netherlands; 8. Faculty of Health, Amsterdam University of Applied Sciences, Amsterdam, The Netherlands.

Corresponding author

Anne M. May, Universiteitsweg 100, 3584CG, Utrecht, The Netherlands;

E-mail: a.m.may@umcutrecht.nl

Phone number: +31887551132

Online Resource 2. Characteristics of the exercise intervention performed in the PACT and PACES trial

	**Timing & duration program**	**Frequency**	**Aerobic training**	**Resistance training**	**Other modalities**
**PACES**	Start at 1st cycle of chemotherapy until 3 weeks after completion therapy	2 times/week	30 min/session, 50% to 80% of maximal estimated workload. Intensity is adjusted using the Borg scale	20 min/session, starting with 2x12 repetitions at 70% of 1RM. Intensity gradually increases to 2x8 repetitions (80% 1RM)	Advice to be physically active 5 days of the week for at least 30 minutes.<br><br>The 'active living' method was used to encourage maintenance of physical activity.
**PACT**	18 weeks, at least partly overlapping with chemotherapy treatment	2 times/week	25 min/session, interval training of alternating intensity. Heart rate at or below ventilatory threshold.	25 min/session, starting with 2x10 repetitions at 65% 1RM, increasing to 1x10 repetitions (75% 1RM) to finally 1x20 repetitions (45% 1RM)	Advice to be physically active 5 days of the week for at least 30 minutes.<br><br>Bandura's social cognitive theory was used to encourage maintenance of physical activity.

Abbreviations: 1RM = one-repetition maximum
