# Supplementary material for: Eight-year follow-up of patient-reported outcomes in patients with breast cancer participating in exercise studies during chemotherapy
Source: J Cancer Surviv. 2024 Aug 5;20(1):123–33. doi: 10.1007/s11764-024-01640-0 (PMC12906584; doi:10.1007/s11764-024-01640-0)
Supplement: Supplementary file 3 — Supplementary file3 (PDF 139 kb) [file 11764_2024_1640_MOESM3_ESM.pdf]

Online Resource 3

Article name

8-year follow-up of patient-reported outcomes in patients with breast cancer participating in exercise studies during chemotherapy

Journal

Journal of Cancer Survivorship

Authors & affiliations

David Binyam[1]/Willeke R. Naaktgeboren[1,2] (shared first), Wim G. Groen[3,4,5], Neil K. Aaronson[2], Anouk E. Hiensch[1], Wim H. van Harten[2,6,7], Martijn M. Stuiver[2,8]/Anne M. May[1] (shared last)

1. University Medical Center Utrecht, The Netherlands; 2. Division Of Psychosocial Research and Epidemiology, The Netherlands Cancer Institute, Amsterdam, The Netherlands; 3. Department of Medicine for Older People, Amsterdam UMC, Vrije Universiteit Amsterdam, Amsterdam, The Netherlands; 4. Aging & Later Life, Amsterdam Public Health Research Institute, Amsterdam, The Netherlands; 5. Amsterdam Movement Sciences, Ageing & Vitality, Rehabilitation & Development, Amsterdam, The Netherlands. 6. Department of Health Services and Technology Research, University of Twente, Enschede, The Netherlands; 7. Rijnstate Hospital, Arnhem, The Netherlands; 8. Faculty of Health, Amsterdam University of Applied Sciences, Amsterdam, The Netherlands.

Corresponding author

Anne M. May, Universiteitsweg 100, 3584CG, Utrecht, The Netherlands;

E-mail: a.m.may@umcutrecht.nl

Phone number: +31887551132

Online Resource 3. Categorized anxiety and depression scores for control and intervention groups at all available timepoints.

	Anxiety		Depression	
	**Control (N=74)**	**Intervention (N=82)**	**Control (N=74)**	**Intervention (N=82)**
**Baseline**				
Case	5 (6.8%)	4 (4.9%)	2 (2.7%)	0 (0%)
Borderline	8 (10.8%)	10 (12.2%)	5 (6.8%)	5 (6.1%)
Normal	61 (82.4%)	68 (82.9%)	67 (90.5%)	77 (93.9%)
**Post-intervention**				
Case	4 (5.4%)	2 (2.4%)	3 (4.1%)	1 (1.2%)
Borderline	4 (5.4%)	7 (8.5%)	8 (10.8%)	6 (7.3%)
Missing	12 (16.2%)	2 (2.4%)	11 (14.9%)	2 (2.4%)
Normal	54 (73.0%)	71 (86.6%)	52 (70.3%)	73 (89.0%)
**6-9 months**				
Case	4 (5.4%)	7 (8.5%)	1 (1.4%)	2 (2.4%)
Borderline	4 (5.4%)	10 (12.2%)	7 (9.5%)	1 (1.2%)
Missing	8 (10.8%)	3 (3.7%)	8 (10.8%)	3 (3.7%)
Normal	58 (78.4%)	62 (75.6%)	58 (78.4%)	76 (92.7%)
**8 years**				
Case	3 (4.1%)	5 (6.1%)	2 (2.7%)	2 (2.4%)
Borderline	5 (6.8%)	8 (9.8%)	4 (5.4%)	6 (7.3%)
Missing	1 (1.4%)	1 (1.2%)	1 (1.4%)	1 (1.2%)
Normal	65 (87.8%)	68 (82.9%)	67 (90.5%)	73 (89.0%)

Anxiety and depression were assessed with the Hospital Anxiety and Depression Scale, with scores of 0-7 corresponding to ‘normal’; 8-10 to ‘borderline case’ and 11-21 to ‘probable case’.
