# Supplementary material for: Eight-year follow-up of patient-reported outcomes in patients with breast cancer participating in exercise studies during chemotherapy
Source: J Cancer Surviv. 2024 Aug 5;20(1):123–33. doi: 10.1007/s11764-024-01640-0 (PMC12906584; doi:10.1007/s11764-024-01640-0)
Supplement: Supplementary file 4 — Supplementary file4 (PDF 264 kb) [file 11764_2024_1640_MOESM4_ESM.pdf]

Online Resource 4

Article name

8-year follow-up of patient-reported outcomes in patients with breast cancer participating in exercise studies during chemotherapy

Journal

Journal of Cancer Survivorship

Authors & affiliations

David Binyam[1]/Willeke R. Naaktgeboren[1,2] (shared first), Wim G. Groen[3,4,5], Neil K. Aaronson[2], Anouk E. Hiensch[1], Wim H. van Harten[2,6,7], Martijn M. Stuiver[2,8]/Anne M. May[1] (shared last)

1. University Medical Center Utrecht, The Netherlands; 2. Division Of Psychosocial Research and Epidemiology, The Netherlands Cancer Institute, Amsterdam, The Netherlands; 3. Department of Medicine for Older People, Amsterdam UMC, Vrije Universiteit Amsterdam, Amsterdam, The Netherlands; 4. Aging & Later Life, Amsterdam Public Health Research Institute, Amsterdam, The Netherlands; 5. Amsterdam Movement Sciences, Ageing & Vitality, Rehabilitation & Development, Amsterdam, The Netherlands. 6. Department of Health Services and Technology Research, University of Twente, Enschede, The Netherlands; 7. Rijnstate Hospital, Arnhem, The Netherlands; 8. Faculty of Health, Amsterdam University of Applied Sciences, Amsterdam, The Netherlands.

Corresponding author

Anne M. May, Universiteitsweg 100, 3584CG, Utrecht, The Netherlands;

E-mail: a.m.may@umcutrecht.nl

Phone number: +31887551132

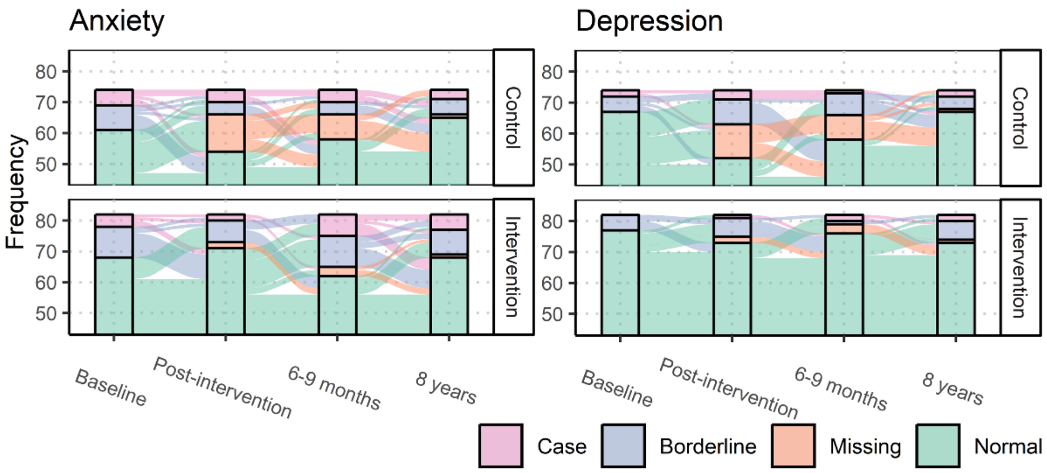

Online Resource 4. Alluvial plots showing the flow of participants for the anxiety and depression categories throughout the study period.

Anxiety and depression were assessed with the Hospital Anxiety and Depression Scale, with scores of 0-7 corresponding to 'normal'; 8-10 to 'borderline case' and 11-21 to 'probable case'. On the y-axis are depicted the number of participants in every category. Note that the graphs are zoomed in to more clearly show the flow of participants in the different categories (as the bottom part of the graph solely consists of participants that are in the 'normal' category and stayed there).
