# Supplementary material for: Eight-year follow-up of patient-reported outcomes in patients with breast cancer participating in exercise studies during chemotherapy
Source: J Cancer Surviv. 2024 Aug 5;20(1):123–33. doi: 10.1007/s11764-024-01640-0 (PMC12906584; doi:10.1007/s11764-024-01640-0)
Supplement: Supplementary file 5 — Supplementary file5 (PDF 322 kb) [file 11764_2024_1640_MOESM5_ESM.pdf]

Online Resource 5

Article name

8-year follow-up of patient-reported outcomes in patients with breast cancer participating in exercise studies during chemotherapy

Journal

Journal of Cancer Survivorship

Authors & affiliations

David Binyam[1]/Willeke R. Naaktgeboren[1,2] (shared first), Wim G. Groen[3,4,5], Neil K. Aaronson[2], Anouk E. Hiensch[1], Wim H. van Harten[2,6,7], Martijn M. Stuiver[2,8]/Anne M. May[1] (shared last)

1. University Medical Center Utrecht, The Netherlands; 2. Division Of Psychosocial Research and Epidemiology, The Netherlands Cancer Institute, Amsterdam, The Netherlands; 3. Department of Medicine for Older People, Amsterdam UMC, Vrije Universiteit Amsterdam, Amsterdam, The Netherlands; 4. Aging & Later Life, Amsterdam Public Health Research Institute, Amsterdam, The Netherlands; 5. Amsterdam Movement Sciences, Ageing & Vitality, Rehabilitation & Development, Amsterdam, The Netherlands. 6. Department of Health Services and Technology Research, University of Twente, Enschede, The Netherlands; 7. Rijnstate Hospital, Arnhem, The Netherlands; 8. Faculty of Health, Amsterdam University of Applied Sciences, Amsterdam, The Netherlands.

Corresponding author

Anne M. May, Universiteitsweg 100, 3584CG, Utrecht, The Netherlands;

E-mail: a.m.may@umcutrecht.nl

Phone number: +31887551132

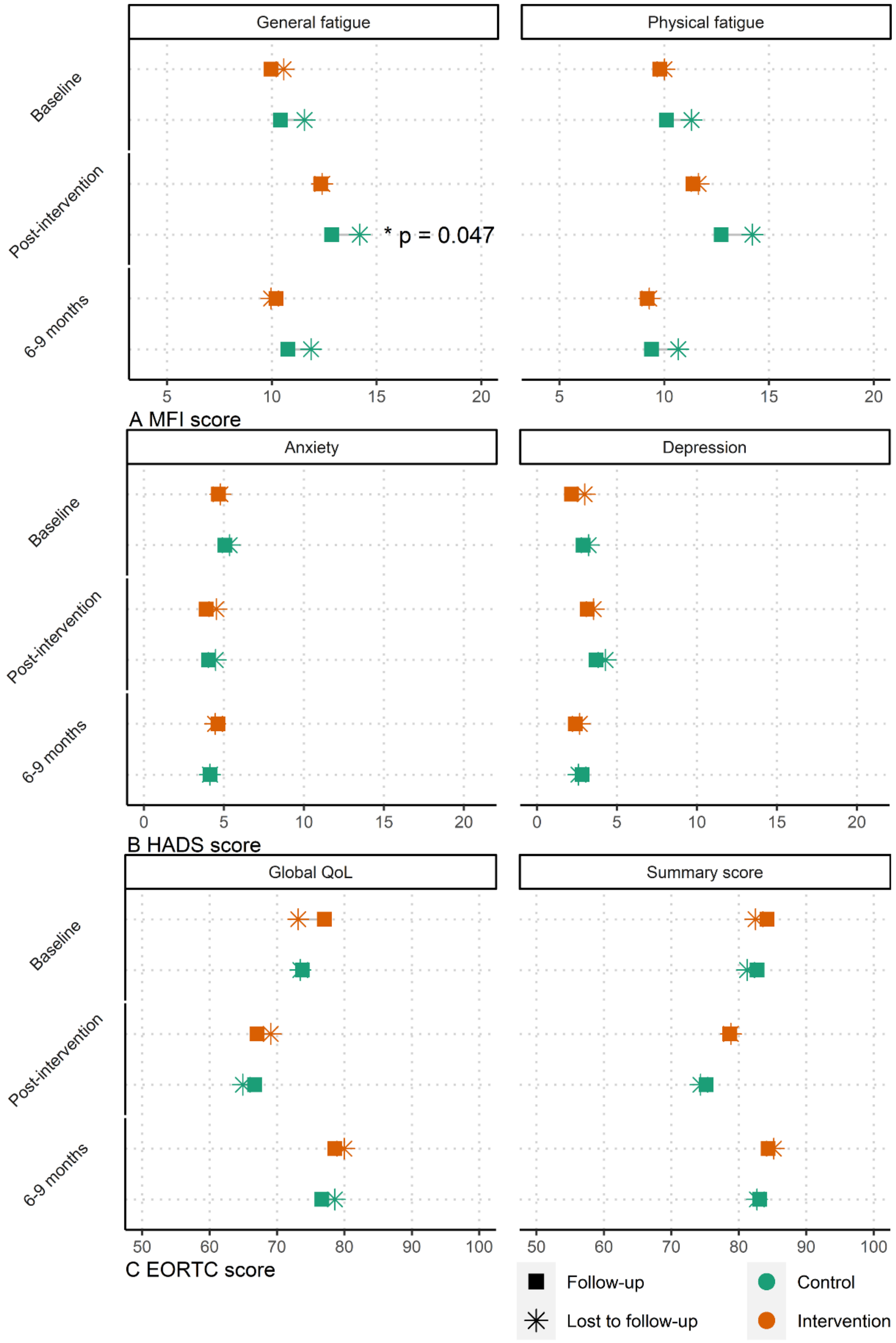

Online Resource 5. Comparison between participants of the PACT and PACES trials, that completed 8-year follow-up and participants that were lost to follow-up.

Mean values at baseline, immediately post-intervention and after 6 to 9 months follow-up for A) general and physical fatigue as assessed by the MFI, B) anxiety and depression as assessed by the HADS and C) global

quality of life and summary score from the EORTC questionnaire. Compared are the intervention (green) and control (orange) groups that completed 8-year follow-up (square) and the groups that were lost to follow-up (star). The between-group differences were tested for significance with an unpaired T-test.

* = significant between-group difference (i.e. $p < 0.05$)

Abbreviations: EORTC = European Organisation for Research and Treatment of Cancer; HADS = Hospital Anxiety and Depression Scale; MFI = Multidimensional Fatigue Inventory; QoL = quality of life
